# Efficient test for nonlinear dependence of two continuous variables

**DOI:** 10.1186/s12859-015-0697-7

**Published:** 2015-08-19

**Authors:** Yi Wang, Yi Li, Hongbao Cao, Momiao Xiong, Yin Yao Shugart, Li Jin

**Affiliations:** 1 Ministry of Education Key Laboratory of Contemporary Anthropology, Collaborative Innovation Center for Genetics and Development, School of Life Sciences, Fudan University, Shanghai, 200433 China; 2 Unit on Statistical Genomics, Division of Intramural Division Programs, National Institute of Mental Health, National Institutes of Health, Bethesda, MD USA; 3 Human Genetics Center, School of Public Health, University of Texas Houston Health Sciences Center, Houston, TX USA; 4 Division of Intramural Research Program, National Institute of Mental Health, National Institute of Health, Porter Bldg, Room 3A100, Bethesda, MD 20892 USA; 5 School of Life Sciences, Fudan University, 2005 Songhu Road, Shanghai, 200433 China

**Keywords:** CANOVA, Linear/nonlinear correlation, Neighborhood, Power, Kidney cancer

## Abstract

**Background:**

Testing dependence/correlation of two variables is one of the fundamental tasks in statistics. In this work, we proposed a new way of testing nonlinear dependence between two continuous variables (X and Y).

**Results:**

We addressed this research question by using CANOVA (continuous analysis of variance, software available at https://sourceforge.net/projects/canova/). In the CANOVA framework, we first defined a neighborhood for each data point related to its X value, and then calculated the variance of the Y value within the neighborhood. Finally, we performed permutations to evaluate the significance of the observed values within the neighborhood variance. To evaluate the strength of CANOVA compared to six other methods, we performed extensive simulations to explore the relationship between methods and compared the false positive rates and statistical power using both simulated and real datasets (kidney cancer RNA-seq dataset).

**Conclusions:**

We concluded that CANOVA is an efficient method for testing nonlinear correlation with several advantages in real data applications.

**Electronic supplementary material:**

The online version of this article (doi:10.1186/s12859-015-0697-7) contains supplementary material, which is available to authorized users.

## Background

Dependence is defined as any statistical relationship between two random variables or sets of data, while correlation describes any of a broad class of statistical relationships, including dependence. In practice, correlation may be useful for indicating a predictive relationship of interest and several methods exist that measure the degree of correlation. The Pearson correlation coefficient is the most commonly used correlation method; however, it is only sensitive to linear correlations, while several other methods tend to be more robust for non-linear correlations [1–3].

The Pearson correlation coefficient (or Pearson’s r), ranging from −1 to 1, was developed by Karl Pearson and was founded on Francis Galton’s related idea [4–8]. Pearson correlation coefficient is defined as the covariance of two variables divided by the product of their standard deviations. Despite the wide use of the Pearson correlation coefficient, there are several negative effects associated with its use, including a non-robust Pearson’s r sample statistic [9], and potentially misleading values in the presence of outliers [10, 11]. The alternative hypothesis for the Pearson correlation test is the linear correlation between two variables X and Y.

The two most common non-linear rank based correlation coefficients are Spearman’s rank correlation coefficient and Kendall’s rank correlation coefficient. Spearman’s rank correlation coefficient (or Spearman’s rho), is a nonparametric measure of statistical dependence between two variables. It is defined as the Pearson correlation coefficient between the ranked variables [12]. The Kendall rank correlation coefficient (or Kendall’s tau coefficient) is used to test the association between two measured quantities [13]. The test is non-parametric, since it does not rely on any assumptions on the distribution of X or Y or (X, Y). The alternative hypothesis for both the Spearman’s correlation test and the Kendall rank correlation test states that the correlation between two variables X and Y corresponds to a monotonic function.

Several other commonly used methods measuring the correlation between random variables include distance correlation, Hoeffding’s independence test, Maximal information coefficient (MIC), Hilbert-Schmidt Information Criterion (HSCI) and Heller Heller Gorfine distance (HHG). The distance correlation is a measure of statistical dependence between two arbitrary variables or random vectors. Distance correlation was introduced by Gabor J Szekely in 2005 to address the deficiency of Pearson’s r (Pearson’s r can be equal to zero for dependent variables) and the initial results on distance correlation were published in 2007 and 2009 [14, 15]. The distance correlation is zero if and only if the random variables are statistically independent. A distance correlation of one implies that the dimensions of the linear spaces spanned by X and Y are almost equal, and Y is a linear function of X. Hoeffding’s independence test (named after Wassily Hoeffding) is a test based on the population measure of deviation from independence. A sample-based version of this measure (as a test statistic) was described with a calculation under the null distribution in 2008 [16]. If the continuous joint distribution and marginal probability densities of two random variables exist, then the Hoeffding’s independence test will be efficient. MIC is a measure of the degree of linear or nonlinear association between two random variables, X and Y. This method is nonparametric and based on maximal information theory [17]. MIC uses binning to apply mutual information to continuous random variables. Binning has been used for applying mutual information to continuous distributions, while MIC is a method for selecting the number of bins and finding a maximum over possible grids. Despite the merits of MIC, there are some limitations of this method as identified by the authors in a later study, specifically that the approximation algorithms with better time-accuracy tradeoffs should be used in computing MIC [18]. The hypothesis of MIC contains a wide range of associations. HSIC (proposed by Gretton et al.) was an independence criterion based on the eigen-spectrum of covariance operators in reproducing kernel Hilbert spaces (RKHSs), consisting of an empirical estimate of the Hilbert-Schmidt Independence Criterion [19]. HHG (proposed by Heller et al.) is a powerful test that is applicable to all dimensions, consistent against all alternatives, and easy to implement [20].

In this work, we focus on the alternative hypothesis that “similar X values lead to similar Y values”, or formally, Y = f(x) + e, e ~ N(0, s), s > 0, and f is a non-constant smooth function. We propose a novel nonlinear correlation measure method: Continuous Analysis of Variance Test (CANOVA). The idea roots in the traditional Analysis of Variance (ANOVA) of continuous response with a categorical factor [21]. ANOVA tests whether the variance within/between categories is smaller/greater than random expectation. For continuous response with continuous factors, we need a generalization of the “within category variance” in ANOVA. In CANOVA, we first define a neighborhood of each data point according to its X value, and then calculate the variance of the Y value within the neighborhood. Finally, we perform a permutation test for the significance of the observed “within neighborhood variance”. We first compare the performance of our CANOVA with six other methods in a simulated dataset. Then we analyze the false positive rate [22] and the statistical power [23] of CANOVA and that of the six other methods on both simulated and real datasets (RNA-seq data on kidney cancer [24, 25]).

## Methods

Given two random variables X and Y, we denote X_i_ and Y_i_ for the *i*th observation. We define the within neighborhood sum square statistics as:

1 $$ \begin{array}{ccc}\hfill \mathrm{W}={\displaystyle \sum_{i,j}}{\left({Y}_i-{Y}_j\right)}^2,\hfill & \hfill j<i,\hfill & \hfill \left| rank\left({X}_i\right)- rank\left({X}_j\right)\right|<K\hfill \end{array} $$

where K is an integer constant provided by the user. Note that |*rank*(*X*_*i*_) − *rank*(*X*_*j*_)| < *K* defines the neighborhood structure of the dataset. The hypothesis of CANOVA is that “similar/neighbor X values lead to similar Y values”. Thus when X and Y are correlated, the W statistics tends to be smaller than random expectation. To evaluate the significance of observed W, we perform a permutation test [26]. When X has equal values (tie), we randomly shuffle the rank of tied X values in each permutation. In a tie situation, for example, with the data: X = 1, 1, 2, 3; Y = 2, 1, 7, 4. Since X has two ones, the sorting of data points is not unique. The algorithm randomly chooses one of the following sorting patterns: X = 1, 1, 2, 3; Y = 2, 1, 7, 4. or X = 1, 1, 2, 3; Y = 1, 2, 7, 4. This algorithm is now implemented by the CANOVA software in Linux system (which is available at https://sourceforge.net/projects/canova/). The CANOVA algorithm (pseudo-code) is summarized as follows:
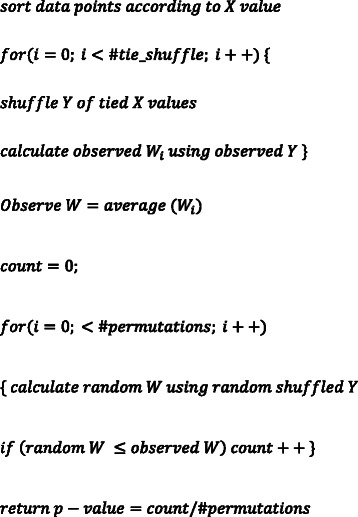


While calculating W, we take advantage of the fact that X_i_ is sorted. Therefore, the algorithm complexity is O(nlogn + np), where n is the sample size and p is the number of permutations. While testing many X variables against one Y variable, we need to do only one permutation of Y and we can reuse the permutation results for all X variables.

### Simulation study

We simulated nine simple functions and added the Gaussian noise (mean = 0, variance = 1) to the Y value for each of them, as shown in Table 1. These included constant functions (i.e. a linear function of the form y = b, where b is a constant, and b = 0 in Table 1 accordingly), linear functions, quadratic functions, sine functions and cosine functions. We varied the Gaussian noise levels (mean = 0, variance = 1/9, 1/4, 4 and 9) in our simulations and reported the power across noise levels (shown in Additional file 1). We benchmarked six methods including the Pearson correlation coefficient, the Spearman’s rank correlation coefficient, the Kendall’s rank correlation coefficient, the Distance correlation, the Hoeffding’s independence test and the Maximal information coefficient. The simulation was repeated 1000 times to calculate the false positive rate and the statistical power. We chose 50 as the sample size (*N* = 50), *x* as the independent variable which was uniformly distributed in (−1, 1) and *y* as the dependent variable. As K is the only parameter of our CANOVA, we assign its value from the positive integer collection (K = 2, 4, 8, 12). Notably, MIC also has a bias/variance parameter (‘alpha’ parameter in the minerva implementation): the maximal allowed resolution of any grid [17]. Reshef et al. [18] also found that the different parameter setting (α = 0.55, c = 5) is faster (than default) and does not appear to significantly affect the performance. For simplicity, here we just used the default parameters (α = 0.6, c = 15) of MIC.

**Table 1:** Simulation power in nine simple functions

N = 50, x ~ U(−1,1)	CANOVA2	CANOVA4	CANOVA8	CANOVA12	Pearson	Kendall	Spearman	Distance	Hoeffding	MIC
y = 0 + N(0,1)	0.051	0.048	0.048	0.050	0.047	0.048	0.049	0.039	0.059	0.051
y = x + N(0,1)	0.564	0.798	0.889	0.902	**0.972**	0.962	0.961	0.950	0.953	0.591
y = 0.5;*(x + 1)^2^ + N(0,1)	0.606	0.836	0.904	0.918	**0.968**	0.953	0.962	0.964	0.953	0.633
y = sin(Pi*x) + N(0,1)	0.758	0.941	0.966	0.962	0.936	0.918	0.930	**0.969**	**0.969**	0.829
y = sin(2*Pi*x) + N(0,1)	0.713	**0.886**	0.812	0.294	0.318	0.328	0.320	0.341	0.405	0.579
y = sin(3*Pi*x) + N(0,1)	0.677	**0.796**	0.254	0.076	0.178	0.192	0.199	0.186	0.219	0.423
y = cos(Pi*x) + N(0,1)	0.784	0.940	**0.973**	0.942	0.067	0.076	0.083	0.660	0.710	0.660
y = cos(2*Pi*x) + N(0,1)	0.738	**0.891**	0.754	0.142	0.045	0.054	0.053	0.100	0.129	0.548
y = cos(3*Pi*x) + N(0,1)	0.673	**0.751**	0.160	0.031	0.053	0.054	0.057	0.074	0.090	0.371

The bold means the first place result of all methods compared

### Applications on real dataset

We applied our proposed CANOVA method to a RNA-seq kidney cancer dataset, and compared the results generated by the other six methods. The kidney cancer data set consists of 604 samples and 20,531 genes [24, 25]. We tested the correlation between genotype data X (20,531 gene-expression data) and phenotype data Y (kidney cancer or not). The computing time of each method was documented for comparison. The significance is preset as 2.435342e-06 (Bonferroni correction). We used an X-Y plot and a grid search (Such as K = (10, 20, 30, 40, 50)) to choose the best K (K = 30) for CANOVA by their corresponding statistical power). For simplicity, the other methods were used the default parameters (especially for MIC, α = 0.6, c = 15). The results and comparisons are shown in Table 2.

**Table 2:** Power comparison in kidney cancer dataset (The significance level α = 0.05/20531)

Kidney cancer dataset	CANOVA	Kendall	Pearson	Spearman	Hoeffding	Distance	MIC
Significant gene number	5901	11569	8239	**11629**	4953	10946	8081
Computing time (seconds)	**24**	65	32	32	44	~10^6^	114

The bold means the first place result of all methods compared

“~” means about or approximately

## Results

### Results from simulation study

As indicated in Table 1, when the constant function (y = 0) was used, we compared the false positive rate of different methods with alpha = 0.05 (significance level). CANOVA with different K (CANOVA2, CANOVA4, CANOVA8 and CANOVA12), the Pearson correlation coefficient, the Spearman’s rank correlation coefficient, the Kendall’s rank correlation coefficient and the Maximal information coefficient all show a false positive rate around 0.05, indicating that the results are correct. Nevertheless, the Distance correlation’s false positive rate is slightly lower than 0.05 and the Hoeffding’s independence test’s false positive rate is a little greater than 0.05. Therefore, it is crucial to note that the significant variables by the Hoeffding’s independence test may be false positives and the true significant variables could be not detected by the Distance correlation.

For power comparison on the non-constant correlations shown in Table 1, we observed the following: (1) when the correlation is linear, the Pearson correlation coefficient is the most powerful. The CANOVA test is less powerful than the Pearson correlation coefficient, but does not fail (power >0.5); (2) In non-linear correlation case, the CANOVA tests are the best, especially when the correlation is highly oscillating/non-linear; (3) The power CANOVA4 is the best single non-linear test, and it is more powerful than MIC with sine and cosine functions.

For our power comparison on the non-constant correlations shown in Additional file 1, we have the following results: (1) when the Gaussian noise levels were low (Gaussian variance = 1/9, 1/4), most methods had higher power especially in simple linear relationships, and the CANOVA (CANOVA2 and CANOVA4) are still among the best methods with the highest power in most non-constant functions; (2) when the Gaussian noise levels were high (Gaussian variance = 4, 9), most methods had lower power while the CANOVA4 had higher power than other methods in complex sine/cosine functions. Nevertheless, the Pearson correlation coefficient and Hoeffding’s independence test presented higher power in simple linear relationship functions. Therefore, when the correlation between two random variables is linear, we recommend using the Pearson correlation coefficient for greater statistical power. When the correlation is nonlinear or complicated, CANOVA with suitable parameter K is a good choice to explore the correlation structure of the data.

### Results from the Kidney Cancer Study

**Fig. 1:**
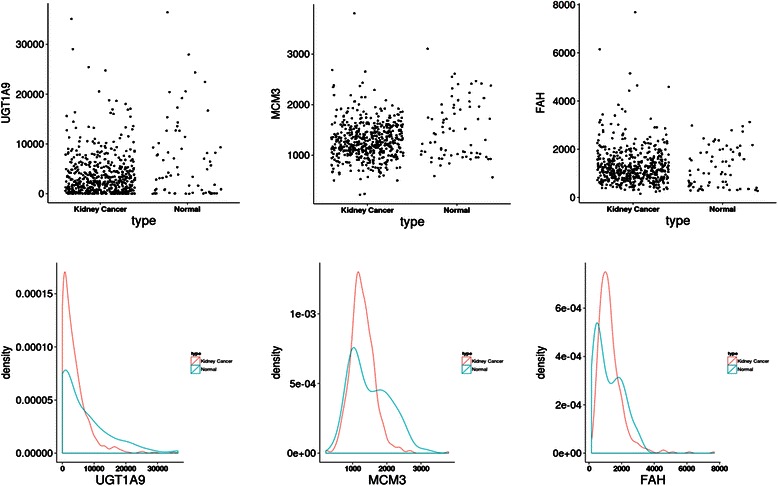
The Scatterplot and probability density distribution of three gene expressions (FAH, MCM3 and UGT1A9) between kidney-cancer and normal groups

The power comparison and computing time for kidney cancer dataset [24, 25] is shown in Table 2. For the purpose of computing time comparison, the number of permutations of CANOVA is set as 10,000,000 (Table 2). We provided in Table 3 the genes only detected by the CANOVA method (that is not detected by other methods, the number of permutations of CANOVA is 100,000,000 in Table 3). For comparison, we also listed the genes only detected by other methods in Additional file 2. To further explore the relationships identified only by CANOVA, the Scatterplot and probability density distribution of gene expressions between case and controls are shown in Fig. 1. All of our CANOVA results were realized in the C++ [27] environment and the benchmarked six methods were calculated by R package ‘energy’ [28], ‘Hmisc’ [29] and ‘minerva’ [30]. All CANOVA results were parallelly (fully using all 8 CPU cores) calculated using a desktop PC, equipped with an AMD FX-8320 CPU and 32GB memory. Additionally all of the R code was parallelly computed by the R package ‘snow’ [31].

**Table 3:** Significant genes detected only by CANOVA and corresponding *p*-value of all methods in kidney cancer data (α = 0.05/20531)

CANOVA_gene	CANOVA	Distance	Hoeffding	Kendall	Pearson	Spearman	MIC
ACY3|91703	0	4.00E-06	0.47918	0.286872598	0.002263414	0.287245869	0.189931316
AMD1|262	0	4.40E-05	0.08116	0.005927801	0.733642545	0.005833208	0.212586042
AMDHD1|144193	3.40E-07	8.00E-06	0.67325	0.030092326	0.000717698	0.029975253	0.170029851
C17orf37|84299	5.80E-07	5.20E-05	0.04005	3.61E-05	0.417383349	3.24E-05	0.219216883
C21orf57|54059	2.40E-07	4.00E-06	0.04784	6.30E-06	3.99E-05	5.38E-06	0.19141914
CRAT|1384	5.80E-07	8.00E-06	0.32615	0.000160458	3.77E-06	0.000149343	0.196028813
ETV5|2119	0	0.000172	0.42256	0.001755105	0.003401714	0.001702658	0.202086913
***FAH|2184***	0	0.000933998	0.48933	0.153797268	0.457070256	0.153962124	0.212691814
FAM105A|54491	0	2.00E-05	0.72088	0.005901803	7.68E-05	0.005807373	0.198623556
FTL|2512	0	0.002467995	0.4743	0.048315704	0.23060211	0.048231442	0.212746271
GDA|9615	1.60E-07	0.00025	0.48634	0.160122916	0.459724584	0.160300937	0.185681164
HSD17B14|51171	0	8.20E-05	0.19284	0.001051631	0.006799576	0.001012728	0.208298029
LOC100132111|100132111	1.00E-08	1.40E-05	0.08222	0.001103681	0.357830837	0.001063627	0.20892751
***MCM3|4172***	0	6.00E-06	0.50714	0.033769054	1.98E-05	0.033657199	0.197222887
MSL3L2|151507	5.00E-08	4.00E-06	0.0658	0.00022671	0.000309573	0.000212513	0.197191821
NPEPPS|9520	6.30E-07	1.80E-05	0.12107	0.006358039	0.294981611	0.006260864	0.193740442
RASEF|158158	4.00E-08	2.00E-05	0.15806	0.038695575	0.339964949	0.038592039	0.221013132
RASGRF1|5923	4.50E-07	0.000509999	0.29384	0.005697491	0.944454242	0.005604368	0.192676281
SLC9A3R1|9368	0	6.00E-06	0.2351	0.001772375	0.000600274	0.001719639	0.211044758
SRGAP2|23380	1.49E-06	1.60E-05	0.13479	0.00010228	0.00076986	9.43E-05	0.16085031
SYTL2|54843	9.40E-07	0.000357999	0.49524	0.156725188	0.013347293	0.156896177	0.197737514
***UGT1A9|54600***	5.00E-08	1.60E-05	0.5995	0.278490278	1.33E-05	0.278854528	0.18041022
ZNF280B|140883	6.80E-07	0.000431999	0.17067	0.073453284	0.259146202	0.073428429	0.203602346
ZNF577|84765	0	4.60E-05	0.13197	0.063783754	0.410213566	0.063735193	0.208902832

As the *p*-value of mic is calculated by table lookup, so we just list the MIC value (If MIC >0.22378, then the *p*-value of MIC < 2.435342e-06)

The genes reported in pubmed was shown in bold italics

Using the kidney cancer RNA-seq data, we indicated in Table 2 that the Spearman method detected the greatest number of significant genes (α = 0.05/20,531), and CANOVA was the fastest method using a desktop PC (equipped with an AMD FX-8320 CPU and 32GB memory). To further explore the biological relevance of the detected genes and to compare the features of each method, we use the uniquely “significant” genes detected from each method as the target gene set, and then performed a literature review for validation of each gene. The uniquely significant genes detected only by CANOVA and the corresponding *p*-value of all methods are shown in Table 3, and the genes reported in pubmed (simply indicating that there is an abstract in pubmed concerning a relationship with kidney cancer and the gene) are shown in bold italics. Similarly, the uniquely significant genes of other methods are shown in Additional file 2.

From the unique set of genes detected by CANOVA (Table 3), a few were reported to be relevant to kidney cancer/disease: FAH, MCM3 and UGT1A9. A defect in FAH results in the accumulation of FAA that can lead to oxidative stress and severe liver and kidney disease [32, 33]. The MCM3 gene was found to be overexpressed in various human cancers, including kidney cancer [34]. The UGT1A9 gene was identified as a major contributor for glucuronidation in the human liver and kidney [35].

From Fig. 1 (MCM3 and FAH), it can be seen that if the normal group distribution is bimodal, and the expression level is mild; an individual is more likely to have kidney cancer. For FAH (Fig. 1), the mean kidney cancer distribution approaches the normal group distribution, which indicates that the linear relationship is almost zero (Pearson R’s *p*-value is about 0.5 in Table 3). Even if the distribution is not bimodal, CANOVA can provide sufficient power if the two distributions have the same mean, but different variances. For example if the control group has a wider distribution (has lower peaks), then it will have thicker tail at the left and right side. This means that higher or lower expression induces protection from the disease, such as in UGT1A9 (Fig. 1).

The only unique gene detected by the Distance method (also reported in Pubmed), IGF1R, is identified in Additional file 2. IGF1R was found to be indirectly associated with kidney cancer tumor growth [36]. Only one gene was detected by MIC (also reported in Pubmed), GIPC2. The GIPC2 gene was reported to be down-regulated in human primary kidney and colorectal tumors [37]. The only unique genes detected by the Pearson method (also reported in Pubmed) was EGR2. The up-regulated EGR2 was found to be involved in overexpressing human embryonic kidney cells, which is indirectly associated with Wilms’ tumors [38]. The only unique gene detected by the Spearman method was COMT. The COMT polymorphism was reported to be associated with renal cell cancer [39]. Alternatively, the Hoeffding and Kendall methods did not detect any unique genes.

## Discussion

CANOVA can be viewed as an extension of ANOVA for continuous variables. We define a neighborhood first and calculate the within neighborhood variance, which is analogous to ANOVA’s within treatment variance. The proposed hypothesis (alternative hypothesis) of CANOVA is that “similar X values lead to similar Y values”. By calculating the variance of Y values of similar/neighbor X values, we are able to test this hypothesis against the null hypothesis.

Local regression [40] is closely related to CANOVA, since both estimate the local residual. Thus, the statistical power would be expected to be similar. For instance, suppose we take a moving average of every K point and then compute the R^2^ between the estimated regression function and the data. Under this condition, two issues would need to be considered: (1) when K is an even number, we need a special treatment of the regression expectation on each data point. (2) On the boundaries data points, some special treatment is required to calculate the unbiased regression expectation. K nearest neighbor (kNN) regression [41] is another type of local regression analogous to CANOVA. CANOVA uses a parameter K to define the neighborhood of data points, while kNN also uses a parameter K to define the nearest neighbor of each data point. CANOVA tests the fitness of the neighborhood model, which is similar to the kNN model. Since Pearson’s correlation coefficient can be viewed as the model fitness test of a linear regression model, CANOVA can be viewed as an analogy of the model fitness test of the kNN model. Using CANOVA, we can conduct the permutation of one Y variable only and perform association tests against many (eg. 20,000) X variables quickly, as the neighborhood structure is independent with X variables. In the case of kNN, the neighborhood structure generated by each X variable is different; therefore, we have to perform a permutation test on every combination of X and Y, which may make kNN slower than CANOVA. Furthermore, CANOVA has the unique advantage of going directly independence testing rather than the unnecessary regression step. Since, we do not need to accurately estimate the regression function at the boundaries, our CANOVA is more theoretically simple and elegant. Based on the aforementioned reasons, we prefer the CANOVA style to local regression style.

The distribution of the W statistics is unknown to us. In the simplest case, where $$ \begin{array}{cc}\hfill \mathrm{K}=2,\hfill & \hfill Y\sim N\left(0,1\right)\hfill \end{array} $$ and *W*_2_ = ∑_*i* >1_(*Y*_*i*_ − *Y*_*i* − 1_)^2^ we know that mean(*W*_2_) = 2N − 2 and var(*W*_2_) = 12N − 16 (calculated by Maple), where *N* is the sample size. Thus, W does not follow any familiar distribution. We had to use a permutation test to assess its significance level. It takes only several seconds for several hundred samples and 10^6^ permutations on a desktop PC, equipped with an AMD FX-8320 CPU and 32GB memory. It can be seen from Table 2 that CANOVA is even faster than Pearson correlation when testing correlation between thousands of features and one response variable Y. The faster speed is due to three reasons: (1) CANOVA is implemented in efficient C++ code, while the Pearson correlation is implemented in relatively slow R (2) CANOVA is paralleled and fully uses all CPU cores, which results in an 8X speed up on our AMD 8 core CPUs. (3) When testing 20,000 X variables against one Y variables, we only need to conduct one permutation test on the Y, and we then can reuse the permutation results for all X variables. Thus, the computational complexity is O(np+#Xnlog(n)), where p is the number of permutations, #X is the number of X variables and n is the sample size. This makes our framework potentially useful for big data.

CANOVA requires a parameter K before performing the test. It is the user’s decision to pick a reasonable K. A larger K has more power on slow-varying functions, while a smaller K has more power on quick-oscillating functions depending on the data. Thus, the user needs some prior knowledge of the function being tested. An X-Y plot will be useful before testing. We suggest a choice of K = SampleSize/20. In practice, we first preset a significant level (0.05/feature numbers), we then use a grid search (such as K = (2, 20, 40, 80, 100, 200)) to choose the best K by their corresponding statistical power. On the other hand, one could also use other methods such as Pearson and MIC to get a better feel of a dataset and choose a reasonable K for CANOVA.

CANOVA and MIC can both be used to test nonlinear correlation; however, CANOVA has its own advantages. While MIC tests all types of non-random correlations, CANOVA tests the alterative hypothesis that “similar X values lead to similar Y values”. Formally, CANOVA’s hypothesis is $$ \begin{array}{ccc}\hfill \mathrm{Y}=\mathrm{f}\left(\mathrm{X}\right)+\mathrm{e},\hfill & \hfill \mathrm{e}\sim \mathrm{N}\left(0,\mathrm{s}\right),\hfill & \hfill \mathrm{s}>0\hfill \end{array} $$ and f is a non-constant smooth function. If the relationship of X and Y can’t be written as Y = f(X) then CANOVA may fail. For example, for a relationship *X* ^2^ + *Y* ^2^ = 1, CANOVA fails and MIC still works. The major purpose of CANOVA is to offer a test of independence. The maximal information coefficient is primarily a measure of effect size, and gives similar scores for relationships of similar strength regardless of relationship type [17]. Measures of effect size can be used to test for independence (using a null hypothesis of zero effect size), but the reverse is not true. Nevertheless, Justin B. Kinney & Gurinder S. Atwal indicate that MIC does not have the property of “equitability”, and the reported simulation evidences contain artifacts [42]. However, Reshef et al. [43] and Murrell et al. [44] have called Kinney and Atwal’s methodology into question. Their work led to the better understanding of equitability and MIC and allowed researchers in the area to move forward.

The CANOVA method is less powerful than the Pearson’s correlation coefficient in the case of linear correlation. This can be viewed as a trade-off between the hypothesis space and statistical power. Pearson’s correlation coefficient has a very narrow alternative hypothesis space (linear correlation), while CANOVA’s alternative hypothesis is more general: $$ \begin{array}{ccc}\hfill \mathrm{Y}=\mathrm{f}\left(\mathrm{X}\right)+\mathrm{e},\hfill & \hfill \mathrm{e}\sim \mathrm{N}\left(0,\mathrm{s}\right),\hfill & \hfill \mathrm{s}>0\hfill \end{array} $$. In practice, many correlations are linear or approximately linear, which makes Pearson, Spearman or Kendall correlation coefficient powerful.

The results of our kidney cancer correlation analysis identified that (Table 3 and Additional file 2), although CANOVA did not detect the largest number of significant unique genes, it found the largest number (three) of genes which were also identified as relevant to kidney cancer in the literature.

The results of three gene expressions distribution (FAH, MCM3 and UGT1A9) indicated that CANOVA could exactly detect the special non-linear relationships (Fig. 1 and Table 3), which other methods could not easily find. These three genes were also reported to be involved in the kidney cancer development process in the literature [32–36].

While each method has its own advantages, the results of different methods can often be correlated. Our simulation results indicate that using both linear correlation coefficient (Pearson, Spearman or Kendall) and non-linear correlation coefficient (CANOVA, MIC, Hoeffding, or Distance) could increase the odds of detecting real biological signals. To conclude, CANOVA appears to be efficient in testing nonlinear correlation and has its own advantages in real data applications.

## Availability of supporting data

The kidney RNA-seq dataset were downloaded from the TCGA datasets (level 3 in TCGA datasets, http://cancergenome.nih.gov/).

## Additional files

Additional file 1:
**The power comparison of simulation study across Gaussian noise levels (mean = 0, variance = 1/9, 1/4, 4 and 9).** (XLSX 11 kb)
Additional file 2:
**The significant (associated with kidney cancer) genes only detected by other methods (not including CANOVA).** (XLSX 56 kb)

## Abbreviations

CANOVA: Continuous analysis of variance
RNA-seq: Ribonucleic acid Sequencing
ANOVA: Analysis of Variance
MIC: Maximal information coefficient
PC: Personal computer
AMD: Advanced Micro Devices
CPU: Central Processing Unit
FAH: Fumarylacetoacetate hydrolase
FAA: Fumarylacetoacetate
MCM3: Minichromosome maintenance 3
UGT1A9: Uridine diphosphate-glucuronosyltransferase 1A9
IGF1R: Insulin-like growth factor 1 receptor
GIPC2: GIPC PDZ domain containing family, member 2
EGR2: Early growth response 2
COMT: Catechol-O-methyltransferase
kNN: k nearest neighbor
TCGA: The Cancer Genome Atlas
